# Ashwagandha: Optimizing the Extraction and Electrospun Nanofiber Production

**DOI:** 10.3390/pharmaceutics17010061

**Published:** 2025-01-05

**Authors:** Maciej Jaskólski, Magdalena Paczkowska-Walendowska, Andrzej Miklaszewski, Judyta Cielecka-Piontek

**Affiliations:** 1 Department of Pharmacognosy and Biomaterials, Poznan University of Medical Sciences, Rokietnicka 3, 60-806 Poznan, Poland; jaskolski.mj@gmail.com (M.J.); jpiontek@ump.edu.pl (J.C.-P.); 2 Faculty of Mechanical Engineering and Management, Institute of Materials Science and Engineering, Poznan University of Technology, 60-965 Poznan, Poland; andrzej.miklaszewski@put.poznan.pl

**Keywords:** ashwagandha extract, antioxidant properties, anti-inflammatory properties, electrospun nanofibers, dissolution, permeability

## Abstract

**Background/Objectives:** This study explores the development of electrospun nanofibers incorporating bioactive compounds from *Withania somnifera* (Ashwagandha) root extract, focusing on optimizing extraction conditions and nanofiber composition to maximize biological activity and application potential. **Methods:** Using the Design of Experiment (DoE) approach, optimal extraction parameters were identified as 80% methanol, 70 °C, and 60 min, yielding high levels of phenolic compounds and antioxidant activity. Methanol concentration emerged as the critical factor influencing phytochemical properties. Electrospinning technology was employed to produce nanofibers using polyvinylpyrrolidone (PVP) and hydroxypropyl-β-cyclodextrin (HPβCD) as carriers, ensuring encapsulation, stabilization, and an enhanced bioavailability of the active compounds. **Results:** Nanofibers demonstrated a high surface-to-volume ratio, rapid dissolution, and significant mucoadhesive properties, making them suitable for oral mucosal applications. The optimal nanofiber composition was determined to be 2.5 mL extract, 25% PVP, and an extract-to-HPβCD ratio of 1:0.6. Statistical modeling confirmed that the electrospinning process did not compromise the antioxidant or anti-inflammatory properties of the extract, with extract content being the primary determinant of biological activity. **Conclusions:** These findings highlight the potential of integrating advanced extraction techniques with nanotechnology to develop innovative delivery systems for traditional herbal remedies. The developed nanofibers offer promising applications in pharmaceuticals, cosmetics, and functional foods, paving the way for a scalable and efficient utilization of Ashwagandha bioactives.

## 1. Introduction

Ashwagandha (*Withania somnifera*) root is a widely recognized medicinal herb in traditional medicine systems, renowned for its adaptogenic, antioxidant, and anti-inflammatory properties [1]. These bioactivities are primarily attributed to their rich composition of withanolides, phenolic compounds, and other secondary metabolites [2]. Despite its therapeutic potential, the bioavailability of Ashwagandha root extract remains a significant challenge [3,4], limiting its effectiveness in conventional formulations. Addressing this limitation, modern nanotechnology offers innovative solutions to improve bioactive compounds’ solubility, stability, and targeted delivery, enhancing their therapeutic efficacy [5].

Electrospinning has emerged as a cutting-edge technique for fabricating nanofibers with unique physicochemical properties, including a high surface-area-to-volume ratio, uniform morphology, and the ability to encapsulate bioactives for controlled release [6,7]. This method allows for incorporating herbal extracts, such as Ashwagandha root extract, into nanofibers, creating advanced delivery systems suitable for pharmaceutical, nutraceutical, and cosmetic applications. The electrospun nanofibers ensure that the bioactive compounds are protected during processing, and their release can be tailored to specific therapeutic needs [8]. So far, Elsherbini et al. have developed a multifunctional scaffold based on tadalafil (TDF)-containing nanoparticles incorporated into polyvinyl alcohol/*Withania somnifera* extract nanofibers, which allowed for improved wound healing in diabetic rats in terms of reduced inflammation and increased angiogenesis [9].

In this work, polyvinylpyrrolidone (PVP) and hydroxypropyl-β-cyclodextrin (HPβCD) were utilized as matrix components for the fabrication of fast-dissolved nanofibers containing Ashwagandha root extract. PVP exhibits exceptional film-forming capabilities, biocompatibility, and ease of electrospinning, which contribute to the production of uniform nanostructures [10]. Additionally, PVP is known for its mucoadhesive properties, making it an ideal polymer for applications in buccal delivery systems [11]. HPβCD is a co-carrier to enhance the solubility and dispersion of hydrophobic bioactive compounds [12]. By forming inclusion complexes with bioactives, HPβCD promotes homogeneous distribution within the nanofiber matrix and improves the solubility and bioavailability of the encapsulated compounds [13].

This study focuses on two primary objectives: optimizing the Ashwagandha root extraction process and evaluating the production and characterization of electrospun nanofibers containing the optimized extract. The optimized extraction process ensures the highest yield of active compounds, while the electrospinning process preserves the bioactivity and enables efficient delivery. Structural and functional evaluations of the nanofibers include analyses of their morphology, active ingredient release profiles, bioadhesion, and biological activities such as antioxidant and anti-inflammatory properties.

## 2. Materials and Methods

### 2.1. Plant Material

The plant material, Ashwagandha root (*Withania somnifera*), was purchased as a commercially available material from NANGA; country of origin: India; best before date 03/2027; batch number: M-0017572.

### 2.2. Chemicals and Reagents

Withanolide A, withanolide B, withanoside IV, withanoside V, deoxywithastramonolide, and withaferin A (phyproof^®^ Reference Substances) were obtained from Sigma-Aldrich (Poznan, Poland). Carriers such as (2-Hydroxypropyl)-β-cyclodextrin (HPβCD) average Mw ~1460) and polyvinylpyrrolidone (PVP) K30 were supplied from Sigma-Aldrich (Poznan, Poland). Sigma-Aldrich (Poznan, Poland) provided reagents like Folin–Ciocalteu reagent, 2.2-Diphenyl-1-picrylhydrazyl (DPPH), bovine serum, hexadecyltrimethylammonium bromide (CTAB), and hyaluronic acid (HA) for activity assays; KCl, NaCl, K_2_HPO_4_, MgCl_2_, CaCl_2_, and xylitol for dissolution studies, and mucin from the porcine stomach for a mucoadhesive assay. Prisma™ HT buffer. Acceptor Sink Buffer, and GIT lipid solution were obtained from Pion Inc. (Billerica, MA, USA), whereas HPLC grade acetonitrile and water were obtained from Merck (Darmstadt, Germany). High-quality pure water and ultra-high-quality pure water were prepared using a Direct-Q 3 UV Merck Millipore purification system (Burlington, MA, USA).

### 2.3. Optimization of the Ashwagandha Root Extract Extraction Process and Investigation of Its Biological Activity

The Design of Experiments (DoE) method was used to generate A 3-level Box-Behnken plan. As independent variables, the extraction mixture’s temperature, composition, and processing time were selected (Table 1).

The following metrics were selected to evaluate the effectiveness of the extraction process: total phenolic compound content, withanolide content, antioxidant activity (DPPH scavenging assay), and anti-inflammatory activities expressed as the suppression of hyaluronidase activity.

The total content of phenolic components (TPC) was determined by using the method described previously [14]. In short, distilled water (200 µL), Folin–Ciocalteu reagent (15 µL), 20% sodium carbonate solution (60 µL), and extract or gallic acid solution (25 µL) were added to each vial. The plate was shaken for 5 min at 600 rpm and then allowed to sit at room temperature for another 25 min in the dark. The absorbance (Multiskan GO 1510, Thermo Fisher Scientific, Vantaa, Finland) was measured at 760 nm. Milligrams of gallic acid equivalents (GAE) per gram of the plant material were the unit of measurement used to determine the extracts’ phenolic content.

Currently, a high-performance liquid chromatography technique has been developed to measure the concentration of six withanolides (withanolide A, withanolide B, withanoside IV, withanoside V, deoxywithastramonolide, and withaferin A) at the same time. A LiChrospher RP-18 column (5 μm. 250 mm × 4 mm) (Merck, Darmstadt, Germany) served as the stationary phase. With a continuous mobile phase flow of 1.5 mL/min, the mobile phase consisted of formic acid 0.1% (A) and acetonitrile (B) in a gradient flow: 0–7 min, 5–25% B; 7–22 min, 25–45% B; 22–32 min, 45–80% B; 32–35 min, 80–100% B; 35–37 min, 100% B; and 37–40 min, 5% B. The temperature of the column was set at 30 °C. It was successfully detected at *λ*_max_ = 227 nm. The method was validated according to the International Conference on Harmonization Guideline (ICH Q2) regarding the linearity and limits of detection and quantification.

Antioxidant activity was determined using an assay with 2.2-Diphenyl-1-picrylhydrazyl (DPPH), as described previously [14]. A total of 175 μL of a 0.2 mmol/L DPPH solution was continuously mixed with 25 μL of extract for a 30 min dark incubation period. At 517 nm, the absorbance was measured to the blank sample, which consisted of 175 μL of methanol and 25 μL of the extraction mixture (Multiskan GO 1510, Thermo Fisher Scientific, Vantaa, Finland).

The process of hyaluronidase inhibition was ascertained using the previously recognized turbidimetric method [14]. In short, the sample was made by combining an acetate buffer (pH 4.5; 15 µL), a hyaluronidase enzyme (30 U/mL; 25 µL), extract (10 µL), and an incubation buffer (50 mM. pH 7.0 with 77 mM NaCl and 1 mg/mL of bovine albumin; 25 µL). A hyaluronic acid solution (0.3 mg/mL; 25 µL) was added after 10 min of incubation at 37 °C, and the mixture was then incubated for an additional 45 min. The turbidity was measured at 600 nm using a Multiskan GO 1510 (Thermo Fisher Scientific, Vantaa, Finland) following the addition of CTAB (200 µL) and a 10-min room temperature waiting period.

### 2.4. Obtaining Electrospun Nanofibers Containing Ashwagandha Root Extract

The electrospinning process was carried out using the NS + NanoSpinner Plus Electrospinning Equipment (Inovenso Ltd., Istanbul, Turkey). Based on Design of Experiment (DoE) data and the 3^2^ full factorial design experimental plan, the amount of extract, HPβCD, and PVP composition utilized to manufacture the nanofibers was chosen and is shown in Table 2 (amounts per 10 mL of the ethanol solution). The solution flow rate was 2 mL/h, the high voltage was set at 27 kV, and the distance between the syringe and the aluminum foil-covered rotating collector was established at 12 cm. The studies were conducted with a humidity of no more than 40% and at 25 °C.

The parameters used to quantify the DoE process response were process efficiency, withanolide content, the total amount of released bioactive, permeability, and system bioadhesion.

The efficiency was assessed by evaluating the mass of the components used to produce the nanofibers and the mass of the finished nanofibers.

### 2.5. Characterization of the Electrospun Nanofibers—Scanning Electron Microscopy (SEM)

The surface morphology of the nanofiber was observed using SEM. The nanofibers were examined with a Quanta 250 FEG (Hillsboro, OR, USA). FE scanning electron microscope following their gold-palladium sputter coating. The diameter of the nanofibers was assessed using the ImageJ program [15].

### 2.6. Characterization of Electrospun Nanofiber’s Functionality

#### 2.6.1. Withanolide Release and Permeability Assays

The rate of dissolution of withanoside IV integrated into the nanofibers, and its penetration through the membrane systems that mimic the walls of the gastrointestinal tract following the release from the nanofibers, were assessed using the chromatographic technique. After withanolides were incorporated into the structure of the nanofibers, their antioxidant capability was evaluated using the spectroscopic approach.

Electrospun nanofibers were subjected to dissolve tests using an Agilent 708-DS dissolving tool. For stirring, a traditional basket method was employed at 37 ± 0.5 °C and 50 rpm. A total of 30 mL of artificial saliva (pH 6.8), potassium chloride 1.20 g, sodium chloride 0.85 g, dipotassium hydrogen orthophosphate 0.35 g, calcium chloride 0.20 g, xylitol 20.0 g, and water up to 1 L were mixed with nanofibers (1 M HCl was used to bring the pH down to 6.8). An equivalent volume of a temperature-stabilized medium was used in place of liquid samples that were taken at specific intervals. The samples were filtered using a nylon membrane filter with a 0.45 µm mesh size. The amounts of withanoside IV in the filtrated acceptor solutions were determined using the previously described HPLC procedure.

The PAMPA^TM^ (parallel artificial membrane permeability assay) gastrointestinal tract (GIT) assay from Pion Inc. was used to examine the permeability of the withanoside IV encapsulated in the nanofibers via synthetic biological membranes. Donor solutions, which were artificial saliva solutions with a pH of 6.8, were used to dissolve the nanofibers. The acceptor plates were filled with the Acceptor Prisma buffer with a pH of 7.4. After assembling the plates, they were incubated for 15 min at 37 °C with constant stirring at 50 rpm. Every experiment was repeated at least three times. The amount of penetration of the withanoside IV was ascertained using the previously described HPLC approach.

The apparent permeability coefficients (*P_app_*) were calculated from the following equation:

$$P_{app}=\frac{-ln\left(1-\frac{C_{A}}{C_{equilibrium}}\right)}{S\times \left(\frac{1}{V_{D}}+\frac{1}{V_{A}}\right)\times t}$$

where *V_D_*—donor volume, *V_A_*—acceptor volume, *C_equilibrium_*—equilibrium concentration $C_{equilibrium}=\frac{C_{D}\times V_{D}+C_{A}\times V_{A}}{V_{D}+V_{A}}$, *C_D_*—donor concentration, *C_A_*—acceptor concentration, *S*—membrane area, and *t*—incubation time (in seconds).

#### 2.6.2. The Nanofibers’ Activity

The antioxidant activity of the electrospun nanofibers was checked using the DPPH assay, and the anti-inflammatory activity was determined as the ability to inhibit the activity of the hyaluronidase enzyme as described in Section 2.3.

#### 2.6.3. Mucoadhesive Properties

A viscometric method was used to assess the binding strength between mucin and the polymers for bioadhesion. The previously described methodology was used to perform the assessment [13]. The force of bioadhesion *F*, or the enhanced intermolecular frictional force per unit area, was computed as follows:

*F* = (*η_t_* − *η_m_* − *η_p_*) × σ

where *η_t_* is the nanofibers’ viscosity coefficient, and *η_m_* is mucin’s viscosity coefficient. *η_p_* is PVP/HPβCD’s viscosity coefficient, and *σ* is the rate of shear per second.

#### 2.6.4. Cytotoxicity Assay

Human normal skin fibroblasts (Hs27 cells), purchased from the American Type Culture Collection (ATCC, Manassas, VA, USA), were incubated with nanofibers for 24 h. The MTT method was used to measure the vitality of the cells using the previously detailed methods [16].

### 2.7. Statistical Analysis

The Design of Experiment (DoE) and statistical studies were conducted using Statistica 13.3 software. The Shapiro–Wilk test was used to determine whether the data were normal. Duncan’s post hoc tests for multiple comparisons and the ANOVA test were used to examine the differences between the mean values. Group differences were deemed significant at *p* < 0.05. Principal component analysis (PCA) was used to analyze correlations using PQStat Software version 1.8.4.142 (2022).

## 3. Results and Discussion

### 3.1. Optimization of the Ashwagandha Root Extract Extraction Process and an Investigation of Its Biological Activity

The Design of Experiment (DoE) approach is gaining importance in many technological processes, including extraction. In process optimization, DoE identifies critical factors and their interactions, enabling precise control over conditions to achieve desired outcomes [17]. Its application in the extraction of Ashwagandha root extract allows for an efficient examination of parameters like temperature, solvent concentration, and extraction time. This facilitates the development of an optimized, cost-effective, and scalable extraction process while maintaining product quality and bioactive compound yield [18,19]. To assess the efficiency of the extraction process and the influence of input parameters (temperature, solvent concentration, and extraction time), the phytochemical properties of the obtained extracts were assessed.

Firstly, the TCP was assessed (Table 3). Extract no. 12, prepared at 70 °C, in two cycles of 90 min and with 60% methanol, contained the most polyphenols (5.92 ± 0.15 mg GAE/g of plant material). When analyzing the significance of input factors, none had a statistically significant effect on TPC. One can only notice the trend of the impact of temperature on TPC (Figure S1, Supplementary Materials). Hydromethanolic extracts of *W. somnifera* roots were examined by Alam et al. [20], who discovered more increased levels of TPC for leaves than roots at 17.80 mg GAE/g DW and 32.58 mg GAE/g DW, respectively. Dhanani et al. [21] analyzed the TPC in Ashwagandha root extracts obtained by different extraction methods (ultrasound-assisted, microwave-assisted extractions, or supercritical fluid extraction). The highest contents were obtained in the case of a microwave-assisted extraction in ethanol extract (40.96 mg/g GAE) and a supercritical fluid extraction with ethanol (35.93 mg/g GAE). Furthermore, Ganguly et al. [22] found that hydromethanolic extracts of *W. somnifera* roots had higher contents of TPC (97.38 µg GA/mg of extract) than aqueous extracts (53.9 µg GA/mg of extract). Yadav and Rai [23] reported comparable levels of TPC for methanolic extracts of the same plant (52.811 mg GAE/100 g DW). The varying extraction processes and analytical techniques employed in each study may cause the variation in phenolic contents found in ashwagandha samples [24]. Furthermore, it has been proposed that the genotype tested or growth factors such as drought, temperature fluctuations, pollution, UV light, and disease attacks affect the amount of phenolic compounds in plants [25,26]. Furthermore, as previously noted by Tomar et al. [27], who found notable variations among plant parts (stems, roots, and leaves), plant parts are essential for the phenolic composition of the extracted extracts.

**Figure 1:**
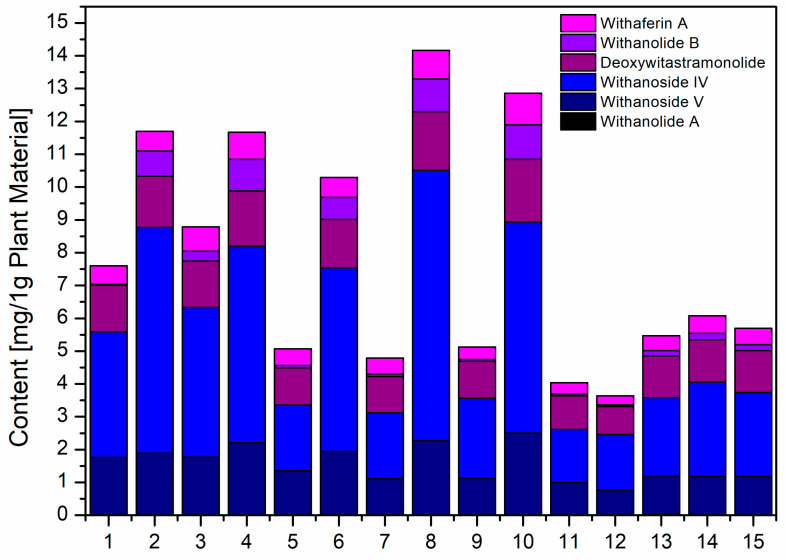
Content of six withanolides in extracts E1–E15.

Based on chromatographic analysis and equations determined for calibration curves for substance standards (Figure S2 and Table S1, Supplementary Materials), the content of active substances in each extract was chosen, as presented in Table 3 and Figure 1. Among the evaluated withanosides, the highest content in the extracts was noted for withanoside IV, which became a quality standard in further studies. By analyzing the above values, it was found that the most significant statistical influence on the average total content of active compounds in the extracts was exerted by the concentration of methanol (Figure S3, Supplementary Materials). This effect has a positive value, which means that increasing the methanol concentration increases the amount of active compounds in the tested extracts.

The antioxidant potential of the tested extracts was evaluated using DPPH radicals, and the results are shown in Table 3. Based on the Pareto chart analysis, it was noted that the methanol concentration has a statistically significant effect on IC_50_ (Figure S4, Supplementary Materials). Antioxidant activity, as tested by Polumackanycz et al. [28], had results for hydromethanolic extracts that ranged from 19.48 to 91.17 mg TE/100 g DW by DPPH assay. Ganguly et al. [22] observed a DPPH value of above 30 µg/mL (IC_50_), whereas Alam et al. [20] reported a DPPH value of 59.16% of inhibition. For aqueous extracts, Polumackanycz et al. obtained results from 93.66 to 281.92 mg TE/100 g DW [28]; the authors also used the ABTS and FRAP methods. The antioxidant activity of *W. somnifera* root aqueous extracts was assessed by Dhanami et al. [21], who found that the DPPH value ranged from 0.40 to 1.20 mg/mL (IC_50_). However, Ganguly et al. [22] got lower levels of DPPH (111.31 µg/mL, IC_50_), whereas Chaudhary et al. [29] reported higher values (4612.17 µg/mL, IC_50_). All these findings from the literature show that the antioxidant activity of *W. somnifera* varies significantly based on the extraction method, and the extraction procedure may also impact the antioxidant potential of natural matrices [30]. Furthermore, because of variations in the amount of withanoside and withanolide, the extraction conditions (such as solvent, extraction duration, and extraction temperature) may impact the antioxidant potential of dried *W. somnifera* leaves and roots [31]. In vitro cell culture, in vivo animal studies, and clinical trials involving healthy individuals have all been used to document Ashwagandha’s antioxidant properties [32,33]. Therefore, Ashwagandha can repair oxidative damage in cells and lipid peroxidation and combat the formation of reactive oxygen species (ROS). It has been demonstrated that the substance withaferin A benefits the neurological system by avoiding cell death and encouraging cell development. Inhibiting the PTEN protein and triggering the PI3K/AKT/mTOR and PI3K/AKT/GSK3β pathways accomplish this. Withaferin A also robustly induces Nrf2, which has cytoprotective properties. Nrf2 activation results in the production of antioxidant proteins, such as heme oxidase-1 (HO-1) and heat shock protein 70 (HSP70), as well as antioxidant enzymes including CAT and SOD, glutathione (GSH), GSH reductase, GPx, thioredoxin (Trx), and Trx reductase [34].

Additionally, the potential for anti-inflammatory action was assessed by assessing the degree of inhibition of the hyaluronidase enzyme activity (Table 3). The extract with the best anti-inflammatory properties was extract no. 10 (2.55 ± 0.09 μg/mL), obtained at 70 °C, in two cycles of 30 min and using 60% methanol as the extractant. Based on the Pareto chart analysis, it was noted that the statistically significant parameter is again methanol. Still, with the increase in its concentration, the anti-inflammatory activity of the extracts decreases (Figure S5, Supplementary Materials). Little information is available in the literature on studies on the inhibition of hyaluronidase activity by Ashwagandha. The results obtained by Chandra et al. [35] showed a concentration-dependent inhibition of protein (albumin) denaturation by Ashwagandha extract. Thus, several different mechanisms may be involved in the anti-inflammatory activity of the plant material. An aqueous solution from Ashwagandha root was found to inhibit the NF-κB and MAPK (mitogen-activated protein kinase) pathways by increasing the expression of anti-inflammatory cytokines and decreasing the expression of pro-inflammatory cytokines such as interleukin (IL)-8, IL-6, tumor necrosis factor (TNF-α), IL-1β, and IL-12 in a study using the HaCaT human keratinocyte cell line [36]. The inhibition of reactive gliosis, production of inflammatory cytokines such as TNF-α, IL-1β, and IL-6, and the expression of nitrooxidative stress enzymes such as iNOS, COX2, NOX2, etc. were observed in mice treated with a water extract from the Ashwagandha. An analysis of NFκB, P38, and JNK MAPK pathways revealed their involvement in the suppression of inflammation, which was further confirmed by inhibitor studies [37].

**Figure 2:**
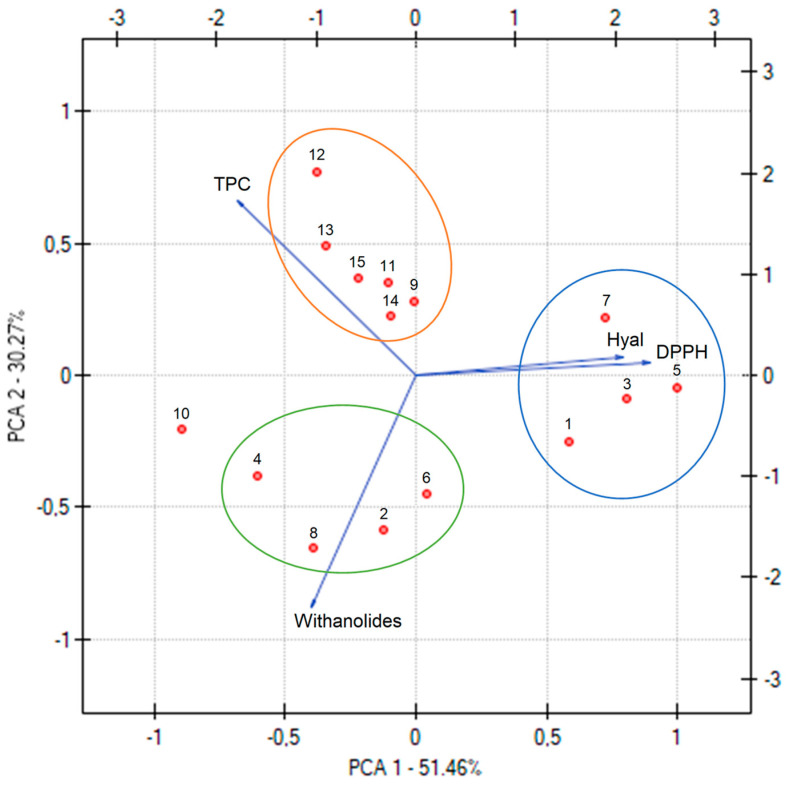
PCA for the phytochemical characterization of extracts, where the next numbers are the numbers corresponding to the extracts.

Figure 2 presents the PCA analysis of the phytochemical properties of all the prepared extracts, revealing key correlations and trends (Table S2, Supplementary Materials). A strong negative correlation is evident between TPC and antioxidant activity, underscoring the critical role of phenolic compounds as potent free radical scavengers—a relationship extensively documented in the literature [38]. This finding highlights the importance of phenolic content in determining the antioxidant efficacy of plant extracts. Interestingly, an inverse correlation is observed between antioxidant and anti-inflammatory activities; as antioxidant activity increases, anti-inflammatory activity tends to decrease, and vice versa. This suggests potential trade-offs between these bioactivities, which the varying chemical composition of the extracts could influence.

Methanol emerges as the most influential factor affecting the phytochemical properties of the extracts, demonstrating its critical role in modulating the extraction efficiency and the yield of bioactive compounds. Furthermore, when the 15 extracts are grouped based on extraction temperature, the samples form three distinct clusters on the PCA graph. These clusters emphasize the pivotal role of extraction temperature in determining the extracts’ phytochemical profile and activity, highlighting the extraction efficiency’s sensitivity to temperature and solvent composition and the resulting bioactive compound profile. This analysis underscores the importance of optimizing extraction conditions, mainly the solvent mixture and temperature, to achieve the desired phytochemical properties and biological activities in Ashwagandha root extracts.

Based on the test results and statistical analyses, the critical technical parameters of the extraction procedure that yielded the extract with optimal qualities and the highest biological activity were successfully identified. Interestingly, the extraction time did not emerge as a statistically significant factor, suggesting that its influence on the extract’s overall extraction efficiency and activity is minimal compared to other parameters. This insight simplifies the optimization process, allowing a focus on more impactful factors. Using the utility contour profiles model, which integrated all the observed outputs into a comprehensive predictive framework, it was possible to forecast and refine the ideal conditions for Ashwagandha root extraction. The optimized parameters included a solvent mixture of 80% methanol, demonstrating superior efficiency in extracting bioactive compounds. The optimal extraction temperature was identified as 70 °C, and the extraction duration was set at 60 min. While the temperature and time were statistically insignificant, their inclusion in the optimized parameters reflects practical and operational considerations. The extract based on the optimized extraction parameters described above was prepared and used for further research (Figure S6, Supplementary Materials).

The optimized extract was tested for total phenolic content (TPC = 4.70 ± 0.10 mg GAE/1 g Plant Material), withanolide content (sum 12.52 ± 0.19 mg/1g Plant Material, including witanolide A 1.62 ± 0.07 mg and withanoside IV 7.22 ± 0.45 mg), antioxidant activity (IC_50_ = 28.55 ± 0.12 µg/mL), and anti-inflammatory effect (12.45 ± 0.12%).

This systematic approach underscores the value of predictive modeling in designing robust extraction protocols, ensuring the maximum yield of bioactive compounds with minimal trial-and-error experimentation. These findings provide a foundation for scaling up the process while maintaining consistency and efficiency in producing Ashwagandha root extracts. Further validation of these conditions in large-scale processes could confirm their applicability and reliability in industrial settings.

### 3.2. Obtaining Electrospun Nanofibers Containing an Ashwagandha Root Extract Characterization of Their Functionality

Electrospinning is a versatile and efficient technique for producing nanofibers with unique structural and functional properties. By incorporating bioactive compounds into these fibers, it is possible to develop advanced materials for applications in pharmaceuticals, cosmetics, and functional foods [39]. Incorporating Ashwagandha root extract into electrospun nanofibers offers an innovative approach to enhancing its bioavailability and controlled release. This process preserves the bioactive compounds and enables their application in diverse fields, providing a new dimension to using traditional herbal remedies in modern technology. This study aimed to assess the influence of nanofiber components on their formation efficiency and their properties relevant to applicability. To produce the nanofibers, polyvinylpyrrolidone (PVP) was selected as a primary polymeric carrier due to its excellent film-forming ability, biocompatibility, and ease of electrospinning into uniform nanostructures [40]. So, does PVP have enough capacity to encapsulate and stabilize bioactive compounds to make it an ideal matrix for incorporating Ashwagandha root extract into nanofibers? Hydroxypropyl-β-cyclodextrin (HPβCD) was employed as a co-carrier due to its remarkable solubilizing properties. HPβCD forms inclusion complexes with hydrophobic bioactive compounds, enhancing their aqueous solubility and bioavailability [41]. So, does its presence ensure the effective encapsulation of Ashwagandha extract while promoting a more homogeneous distribution of bioactive compounds in the nanofiber matrix? It is worth finding an answer to the hypothesis posed: Does the synergistic use of PVP and HPBCD not only facilitate the electrospinning process but also contribute to the structural integrity and functional properties of the nanofibers, ensuring that the encapsulated Ashwagandha extract retains its bioactive potential for targeted applications?

The first step was to evaluate the percentage efficiency of producing nanofibers from starting components (Table 4). No statistically significant effect of any of the components on the mass of the obtained nanofibers was observed (Figure S7, Supplementary Materials). It is worth noting that extreme process conditions (nanofibers no. 1 and 9) cause process disturbances and a very low final product mass.

**Figure 3:**
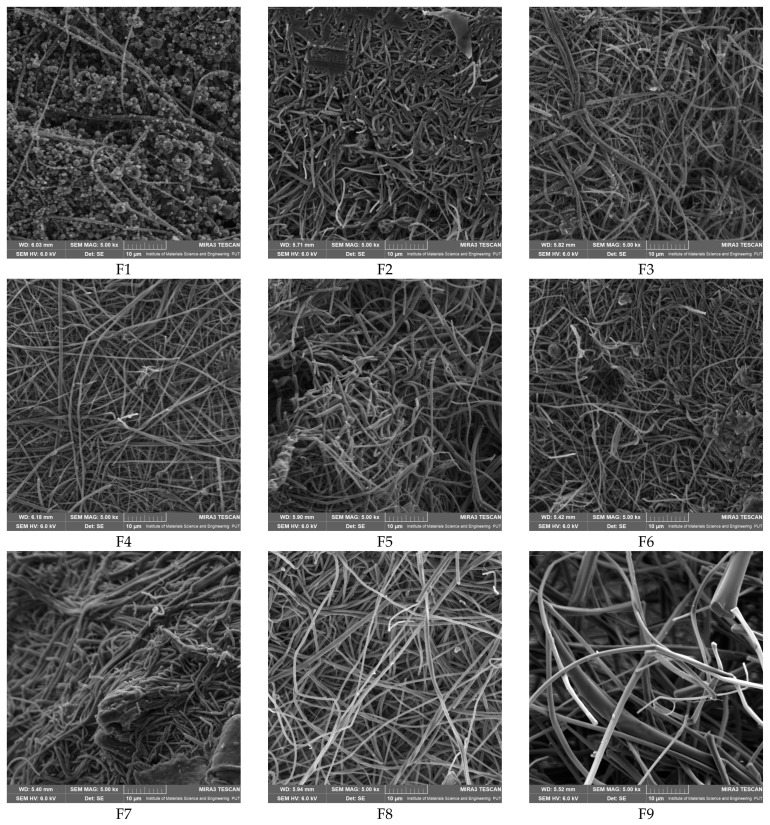
SEM images of electrospun nanofibers F1–F9.

The SEM images in Figure 3 show the morphology of the produced nanofibers. Morphological results show that a homogenous network of the nanofibers with diameters in the 238–508 nm range was obtained (Figure 3, Table 5). Except for nanofiber F1, there are no noticeable structural defects in the fibers; these pictures show the effectiveness of the electrospinning process under the given parameters and circumstances. No statistically significant relationships were observed between the composition and diameter of the nanofibers (Figure S8, Supplementary Materials).

As seen in Table 6, the first element to be looked at was the amount of withanoside IV within the nanofibers. It is important to note that the amount of extract and the extract-to-HPβCD ratio had a statistically significant impact on the substance content when examining the Pareto chart (Figure S9, Supplementary Materials).

**Figure 4:**
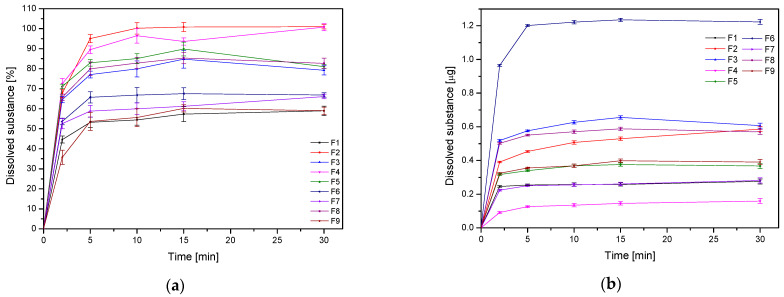
Dissolution profiles of withanoside IV from nanofibers F1–F9, expressed in % (**a**) and µg (**b**) (*n* = 3).

The release of the active ingredient from the nanofibers is a crucial factor that greatly impacts the product’s effectiveness (Figure 4, Table 7). For this purpose, a previously developed, modified method using baskets was used [13]. A rapid-release profile of the active compound is noticeable for all structures. The advantages of producing nanofibers, such as their high surface-to-volume ratio that can quicken the pace of dissolution, great load-bearing capacity, and efficient encapsulation, can explain this phenomenon [42]. When analyzing the Pareto chart, it is worth noting that the amount of extract and the ratio of extract-to-HPβCD had a statistically significant effect on the amount of the released substance (Figure S10, Supplementary Materials). Since the applied HPβCD demonstrated a rise in the solubility of active compounds due to the high amorphizing, wetting, solubilizing, and complexing capabilities of this cyclodextrin [43], an increase in HPβCD content does not have a further solubilizing effect on the active compounds, but its presence is significant.

Also, due to low oral bioavailability and first-pass metabolism [44], the oral mucosa is thought to be a good place to administer withanosides. The rate of the withanoside IV release from electrospun nanofibers was demonstrated to be significantly increased. Permeation through biological membranes may then increase as a result. Thus, the withanoside IV permeability from the produced extract and nanofibers was evaluated using the PAMPA test (Table 8), an in vitro model that assesses passive transport. It is worth noting that the penetration of the compound from the extract and nanofibers is 10 times higher than in the standard case, indicating a significant entourage effect. Analyzing the Pareto chart, it was shown that with the increase in the concentration of the extract and the amount of HPβCD), the compound’s permeability decreases; therefore, the optimization of the composition is essential (Figure S11, Supplementary Materials).

It is worth noting an additional environmental aspect. With the increased bioavailability of active compounds, it is possible to use a smaller amount of extract-containing material to obtain an effective therapeutic dose. This is important from the point of view of non-biodegradable residues and having the ability to accumulate in virtually all environmental media biopolymers [45]. On the other hand, cyclodextrin-based materials in environmental remediation provide an eco-friendly alternative to conventional methods, often involving harsh chemicals or energy-intensive processes. Their application enhances the effectiveness of resource recovery and pollutant removal while lowering the total environmental impact [46].

The acquired data can be extrapolated to evaluate the increase in withanolides’ bioavailability by demonstrating an increase in the rate of withanoside IV release at oral pH, the compound with the highest content in the extract, and an increase in the compound’s permeability across the membrane mimicking the mucosa.

Maintaining the electrospun nanofibers in the oral cavity, namely on the buccal layer, for the exact duration required for the active component to dissolve and penetrate is crucial when assessing the product’s potential for use in the oral cavity. As a result, the resulting electrospun nanofibers’ rheological characteristics were evaluated (Table 9). It is worth noting that PVP is responsible for the increased bioadhesion; as the PVP concentration increases, the degree of mucoadhesion increases (Figure S12, Supplementary Materials). Based on the literature, PVP is appraised for mucoadhesive properties [47]. Membranes containing PVP showed a higher swelling ability and, therefore, a higher mucoadhesion [48].

Finally, using nanofibers with the methods outlined in the article’s first section, we evaluated whether the extract’s anti-inflammatory and antioxidant properties remained (Table 10). Firstly, the results confirmed that this process had no negative impact on the biological activity of the finished nanofibers, as the antioxidant and anti-inflammatory properties remained at the level resulting from the presence of the extract (Figures S13 and S14, Supplementary Materials). Secondly, it was shown that the individual components had no statistically significant impact on the biological activity. Nonetheless, it was evident from the trend that the extract had a considerable effect on the nanofibers’ activity.

**Figure 5:**
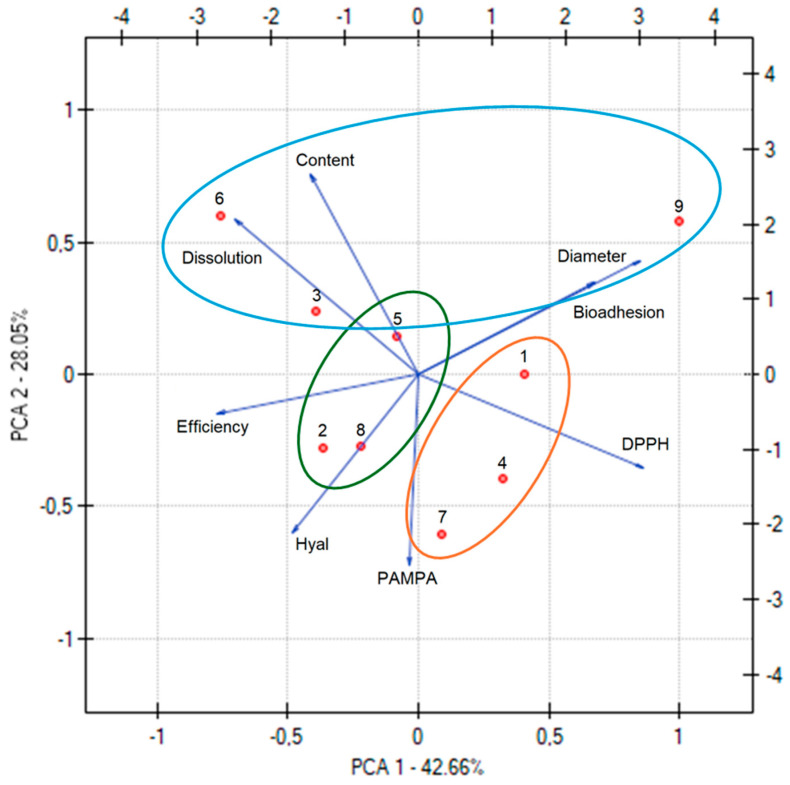
PCA for the characterization of the electrospinning process and obtained nanofibers.

Figure 5 presents the results of the PCA analysis of the nanofibers’ characteristics. A statistically significant strong negative correlation was observed between the diameter of the nanofibers and their production efficiency (Table S3, Supplementary Materials); a higher production efficiency was associated with nanofibers having smaller average diameters. Additionally, a strong correlation was identified between the content of withanoside within the nanofibers and its dissolution. Furthermore, a strong correlation was found between the content of withanoside, its dissolution, and the antioxidant activity of the nanofibers; the greater the amount released, the higher the antioxidant activity.

Analyzing the significance of input factors and their influence on output parameters, a statistical relationship was observed in many cases concerning the extract content. Notably, all nanofibers and their properties can be categorized based on the extract content, represented by the three groups in Figure 5. This demonstrates a significant influence of the extract content on parameters such as the substance content within the nanofibers and its release behavior. For example, this is particularly evident in the group of nanofibers containing 3 mL of the extract (blue line).

The completed research and statistical studies (Figure S15, Supplementary Materials) made it possible to develop a statistical model and identify the optimal composition of the nanofibers. The nanofiber composition’s ideal parameters were 2.5 mL of extract, 25% PVP, and an extract-to-HPβCD ratio of 1:0.6 (for 10 mL of ethanol solution).

The optimized nanofibers were tested for process efficiency (65.89%), withanolide content (0.42 ± 0.01 µg in 100 mg of nanofibers), the total amount of the released bioactive (dissolution of 1.08 ± 0.06 μg in 5 min), permeability (P_app_ 21.12 ± 0.04 × 10^−6^ cm/s), and the systems’ bioadhesion (135 ± 10 cps).

Cytotoxicity was assessed using the MTT assay using normal human skin fibroblast lines (Hs27 cells) to determine the biocompatibility of the obtained materials. Nanofibers at a 100 mg/mL concentration did not interfere with cell fusion (cell viability above 98% compared to the control). The lack of influence of nanofibers on the viability of fibroblast cells proves the biocompatibility of the obtained material.

The findings of this study lay a solid foundation for further exploration into the stability of electrospun nanofiber systems containing Ashwagandha extract. Future research can focus on comprehensive stability studies to ensure these innovative delivery systems’ long-term efficacy and safety. Some key perspectives for advancing the application and understanding of these systems include the following: (1) the physicochemical stability of nanofibers to assess the structural integrity, moisture uptake, and mechanical properties of nanofibers under different storage conditions (e.g., temperature, humidity, and light exposure) including assessing changes in fiber morphology, encapsulation efficiency, and drug release profiles over time; (2) determining shelf life through the use of accelerated stability testing will provide essential data for commercialization; (3) designing optimized packaging solutions that protect nanofibers from environmental factors such as oxygen, light, and moisture will increase their stability; (4) taste and sensory evaluation; and (5) beyond in vitro characterization, as in vivo studies should assess the dissolution and permeability of the nanofibers in biological environments, particularly in buccal delivery systems as well as assessing the efficacy of use in animal models and clinical trials at last. By addressing these aspects, developing stable, scalable, and effective nanofiber systems will advance their application in healthcare, particularly for delivering Ashwagandha’s bioactive compounds in therapeutic contexts. This will pave the way for broader clinical and commercial adoption, ensuring consistent performance and patient benefits.

## 4. Conclusions

This study successfully combined optimizing the Ashwagandha root extraction process with developing advanced electrospun nanofibers for an efficient delivery of bioactive compounds. Critical parameters influencing the extraction process were systematically analyzed using the Design of Experiment (DoE) approach. The optimized conditions—70% methanol as the solvent, 80 °C extraction temperature, and a 60-min duration—maximized the yield of phenolic compounds, withanosides, and other bioactives while maintaining process scalability and cost efficiency.

Incorporating the optimized extract into electrospun nanofibers, utilizing PVP and HPβCD as matrix components, demonstrated the potential for creating innovative delivery systems. PVP provided structural integrity and bioadhesive properties, while HPβCD enhanced the solubility and uniform distribution of hydrophobic bioactives. Characterization of the nanofibers confirmed their uniform morphology, effective encapsulation, and controlled release of active compounds, preserving the antioxidant and anti-inflammatory properties of the Ashwagandha extract.

This study also highlighted the potential of nanofiber-based formulations for applications in buccal delivery systems, where the enhanced bioavailability, increased permeability, and mucoadhesion of the fibers offer significant therapeutic advantages. Notably, the electrospinning process maintained the bioactivity of the extract, demonstrating its suitability for producing functionalized delivery platforms.

## Figures and Tables

**Table 1:** Factorial extraction process experiment plan.

No.	% of Methanol in the Extraction Mixture	Temperature [°C]	Time [min]
E1	30	30	60
E2	90	30	60
E3	30	70	60
E4	90	70	60
E5	30	50	30
E6	90	50	30
E7	30	50	90
E8	90	50	90
E9	60	30	30
E10	60	70	30
E11	60	30	90
E12	60	70	90
E13	60	50	60
E14	60	50	60
E15	60	50	60

**Table 2:** Factorial nanofibers production process experiment plan.

No.	PVP Content [%]	Extract Content [mL]	HPβCD Content [Attitude Towards the Extract]
F1	10	1	1:0.5
F2	10	2	1:1.5
F3	10	3	1:1
F4	20	1	1:1.5
F5	20	2	1:1
F6	20	3	1:0.5
F7	30	1	1:1
F8	30	2	1:0.5
F9	30	3	1:1.5

**Table 3:** Total phenolic content, antioxidant, and anti-hyaluronidase activities of extracts E1–E15 (*n* = 6).

No.	TPC [mg GAE/1 g Plant Material]	Sum of Withanolides [mg/1 g Plant Material]	Antioxidant Activity	Anti-Inflammatory Activity
			DPPH IC_50_ [µg/mL]	Inhibition of Hyaluronidase Activity [%]
E1	3.84 ± 0.21 ^f^	7.71 ± 0.56 ^f^	71.78 ± 2.74 ^h^	26.26 ± 3.02 ^c^
E2	4.05 ± 0.22 ^e,f^	11.96 ± 0.78 ^c^	36.00 ± 0.96 ^c,d,e^	18.82 ± 1.84 ^e,f^
E3	4.47 ± 0.15 ^c,d,e,f^	8.93 ± 0.55 ^e^	63.97 ± 4.77 ^g^	61.39 ± 3.02 ^a^
E4	4.72 ± 0.14 ^c,d,e^	11.99 ± 0.75 ^c^	29.40 ± 0.14 ^a,b^	3.64 ± 0.21 ^h^
E5	3.72 ± 0.17 ^f^	5.17 ± 0.32 ^h^	77.74 ± 6.19 ^i^	41.93 ± 2.45 ^b^
E6	4.05 ± 0.28 ^e,f^	10.53 ± 0.62 ^d^	40.04 ± 0.89 ^e^	23.96 ± 1.67 ^c^
E7	4.39 ± 0.62 ^d,e,f^	4.89 ± 0.15 ^h,i^	97.14 ± 2.68 ^j^	19.96 ± 1.53 ^e^
E8	4.49 ± 0.35 ^c,d,e,f^	14.47 ± 1.27 ^a^	33.86 ± 0.65 ^b,c,d^	15.30 ± 1.06 ^g^
E9	4.86 ± 0.49 ^b,c,d,e^	5.23 ± 0.35 ^g,h^	49.93 ± 3.85 ^f^	17.30 ± 1.77 ^e,f,g^
E10	5.53 ± 0.32 ^a,b^	13.16 ± 0.15 ^b^	28.21 ± 0.64 ^a^	2.55 ± 0.09 ^h^
E11	4.82 ± 0.38 ^b,c,d,e^	4.13 ± 0.26 ^i,j^	39.52 ± 1.23 ^e^	15.64 ± 1.20 ^f,g^
E12	5.92 ± 0.15 ^a^	3.74 ± 0.30 ^j^	31.31 ± 1.50 ^a,b,c^	23.20 ± 2.04 ^c,d^
E13	5.58 ± 0.2 ^a,b^	5.60 ± 0.32 ^g,h^	38.54 ± 2.78 ^d,e^	17.40 ± 0.60 ^e,f,g^
E14	4.95 ± 1.04 ^b,c,d^	6.23 ± 0.55 ^g^	38.42 ± 2.71 ^d,e^	24.01 ± 2.37 ^c,d^
E15	5.27 ± 0.62 ^a,b,c^	5.85 ± 0.51 ^g,h^	38.48 ± 2.53 ^d,e^	20.70 ± 1.49 ^d,e^

Mean values within a column with the same letter are not significantly different at *p* = 0.05 using Duncan’s test.

**Table 4:** The efficiency of the electrospinning process.

No.	Efficiency [%]
F1	7.50
F2	58.74
F3	56.98
F4	24.11
F5	55.12
F6	49.15
F7	53.62
F8	27.82
F9	7.62

**Table 5:** Diameters of nanofibers F1–F9.

No.	Fiber Diameter [nm]
F1	-
F2	320.51 ± 80.88 ^a,b^
F3	503.66 ± 127.29 ^b^
F4	393.77 ± 81.96 ^a,b^
F5	467.03 ± 127.21 ^b^
F6	238.10 ± 56.75 ^a^
F7	375.46 ± 117.42 ^a,b^
F8	412.09 ± 137.60 ^a,b^
F9	1098.90 ± 157.57 ^c^

Mean values within a column with the same letter are not significantly different at *p* = 0.05 using Duncan’s test.

**Table 6:** Withanoside IV content in nanofibers F1–F9 (*n* = 3).

No.	Content [µg] in 100 mg of Nanofibers
F1	0.34 ± 0.02 ^b^
F2	0.18 ± 0.02 ^e^
F3	0.32 ± 0.01 ^b^
F4	0.14 ± 0.01 ^f^
F5	0.27 ± 0.02 ^c^
F6	0.44 ± 0.01 ^a^
F7	0.12 ± 0.01 ^f^
F8	0.20 ± 0.02 ^d,e^
F9	0.21 ± 0.02 ^d^

Mean values within a column with the same letter are not significantly different at *p* = 0.05 using Duncan’s test.

**Table 7:** The total amount of released withanoside IV from nanofibers F1–F9 at 5 min (*n* = 3).

No.	Withanoside IV Dissolution in 5 min [μg]
F1	0.26 ± 0.03 ^e^
F2	0.45 ± 0.02 ^c^
F3	0.58 ± 0.04 ^b^
F4	0.13 ± 0.01 ^f^
F5	0.34 ± 0.02 ^d^
F6	1.20 ± 0.06 ^a^
F7	0.25 ± 0.01 ^e^
F8	0.55 ± 0.02 ^b^
F9	0.36 ± 0.03 ^d^

Mean values within a column with the same letter are not significantly different at *p* = 0.05 using Duncan’s test.

**Table 8:** Apparent permeability coefficients for withanoside IV from nanofibers F1–F9, from extracts as well as the standard (*n* = 6).

No.	P_app_ × 10^−6^ [cm/s]
F1	21.96 ± 0.02 ^a^
F2	20.58 ± 0.04 ^d^
F3	20.48 ± 0.29 ^d^
F4	21.32 ± 0.01 ^b^
F5	20.56 ± 0.09 ^d^
F6	20.67 ± 0.04 ^c,d^
F7	22.01 ± 0.03 ^a^
F8	21.76 ± 0.15 ^a^
F9	19.82 ± 0.20 ^e^
Extract	21.01 ± 0.54 ^b,c^
Standard	1.94 ± 0.15 ^f^

Mean values within a column with the same letter are not significantly different at *p* = 0.05 using Duncan’s test.

**Table 9:** The component of the bioadhesion of nanofibers F1–F9 (*n* = 3).

No.	Component of Bioadhesion (cps)
F1	19 ± 2 ^f^
F2	45 ± 4 ^e^
F3	55 ± 2 ^e^
F4	89 ± 3 ^d^
F5	95 ± 5 ^d^
F6	90 ± 4 ^d^
F7	201 ± 13 ^b^
F8	166 ± 15 c
F9	585 ± 20 ^a^

Mean values within a column with the same letter are not significantly different at *p* = 0.05 using Duncan’s test.

**Table 10:** Biological activity of nanofibers F1–F9 (*n* = 6).

No.	Antioxidant Activity	Anti-Inflammatory Activity
	DPPH IC_50_ [mg nanofibers/mL]	Inhibition of Hyaluronidase Activity [%] at Concentration 200 mg nanofibers/ml
F1	213.01 ± 6.58 ^e,f^	0.01 ± 0.01 ^e^
F2	114.91 ± 5.29 ^c^	2.50 ± 0.25 ^a^
F3	101.14 ± 0.90 ^b^	1.16 ± 0.13 ^c^
F4	216.70 ± 4.78 ^f^	0.78 ± 0.08 ^d^
F5	143.00 ± 4.33 ^d^	0.05 ± 0.01 ^e^
F6	86.60 ± 1.06 ^a^	0.69 ± 0.07 ^d^
F7	207.59 ± 7.23 ^e^	1.73 ± 0.17 ^b^
F8	110.07 ± 3.20 ^c^	2.51 ± 0.24 ^a^
F9	212.22 ± 5.73 ^e,f^	0.01 ± 0.01 ^e^

Mean values within a column with the same letter are not significantly different at *p* = 0.05 using Duncan’s test.

## Data Availability

Data are contained within the presented article or Supplementary Materials.

## Supplementary Materials

The following supporting information can be downloaded at https://www.mdpi.com/article/10.3390/pharmaceutics17010061/s1; Figure S1. Statistical analysis for total phenolic content in extracts E1–E9: (a) Pareto plot of standardized effects for total phenolic content in extracts E1–E15; (b) Response surface plots presenting the dependence of extraction temperature and time on the total phenolic content for constant methanol content in the extraction mixture at level 60%. Figure S2. HPLC chromatogram. Table S1. Validation parameters of HPLC method. Figure S3. Statistical analysis for withanolides content in extracts E1–E15: (a) Pareto plot of standardized effects for withanolides content in extracts E1–E15; (b) Response surface plots presenting the dependence of methanol content in the extraction mixture and extraction temperature on withanolides content for constant time at level 60 min. Figure S4. Statistical analysis for antioxidant activity of extracts E1–E15 measured by DPPH method: (a) Pareto plot of standardized effects for antioxidant activity; (b) Response surface plots presenting the dependence of methanol content in the extraction mixture and extraction temperature on antioxidant activity of extracts for constant time at level 60 min. Figure S5. Statistical analysis for anti-inflammatory activity of extracts E1–E15: (a) Pareto plot of standardized effects for anti-inflammatory activity; (b) Response surface plots presenting the dependence of methanol content in the extraction mixture and extraction temperature on anti-inflammatory activity of extracts for constant time at level 60 min. Table S2. Correlation matrix for phytochemical activities. Figure S6. Prediction of the optimization model for obtaining extracts based on effect with positive sign-like TPC, withanolides content and DPPH (a) and those with negative sign-like hyaluronidase assay (b). Figure S7. Statistical analysis for process efficiency: (a) Pareto plot of standardized effects for viscosities of the prepared solutions for electrospinning; (b) Response surface plots presenting the dependence of PVP and extract content on the efficiency of electrospinning process for HPβCD content at level 1. Figure S8. Statistical analysis for diameter of nanofibers: (a) Pareto plot of standardized effects for diameter of nanofibers F1–F9; (b) Response surface plots presenting the dependence of HPBCD and extract content on the nanofibers’ diameter for constant PVP content at level 20%. Figure S9. Statistical analysis for withanoside IV content: (a) Pareto plot of standardized effects for withanoside IV content in nanofibers F1–F9; (b) Response surface plots presenting the dependence of HPβCD and extract content on the withanoside IV content for constant PVP content at level 20%. Figure S10. Statistical analysis for withanoside IV dissolution: (a) Pareto plot of standardized effects for withanoside IV dissolution in 5 min from nanofibers F1–F9; (b) Response surface plots presenting the dependence of extract and HPβCD content on the withanoside IV dissolution for constant PVP at level 20%. Figure S11. Statistical analysis for withanoside IV permeability: (a) Pareto plot of standardized effects for withanoside IV permeability from nanofibers F1–F9; (b) Response surface plots presenting the dependence of extract and HPβCD content on the withanoside IV permeability for constant PVP content at level 20%. Figure S12. Statistical analysis for component of mucoadhesion: (a) Pareto plot of standardized effects for mucoadhesive properties of nanofibers F1–F9; (b) Response surface plots presenting the dependence of PVP and HPβCD content on the component of mucoadhesion for constant extract content at level 2 mL. Figure S13. Statistical analysis for antioxidant activity of nanofibers: (a) Pareto plot of standardized effects for antioxidant activity of nanofibers F1–F9; (b) Response surface plots presenting the dependence of extract and HPβCD content on the antioxidant activity for constant PVP content at level 20%. Figure S14. Statistical analysis for anti-inflammatory activity of nanofibers: (a) Pareto plot of standardized effects for anti-inflammatory activity of nanofibers F1–F9; (b) Response surface plots presenting the dependence extract and PVP content on the anti-inflammatory activity for constant HPβCD content at level 1. Table S3. Correlation matrix for nanofibers properties. Figure S15. Prediction of the optimization model for obtaining extracts based on effects with positive sign-like process efficiency, withanoside IV content, dissolution and permeability, Hyal as well as component of mucoadhesion (a) and this with negative sign-like nanofibers diameters and PAMPA (b).
