# Supplementary material for: Ashwagandha: Optimizing the Extraction and Electrospun Nanofiber Production
Source: Pharmaceutics. 2025 Jan 5;17(1):61. doi: 10.3390/pharmaceutics17010061 (PMC11768389; doi:10.3390/pharmaceutics17010061)
Supplement: Supplementary file 1 [file pharmaceutics-17-00061-s001.zip › pharmaceutics-3390420-supplementary.pdf]

Ashwagandha: Optimizing the Extraction and Electrospun Nanofiber Production

Supplementary material

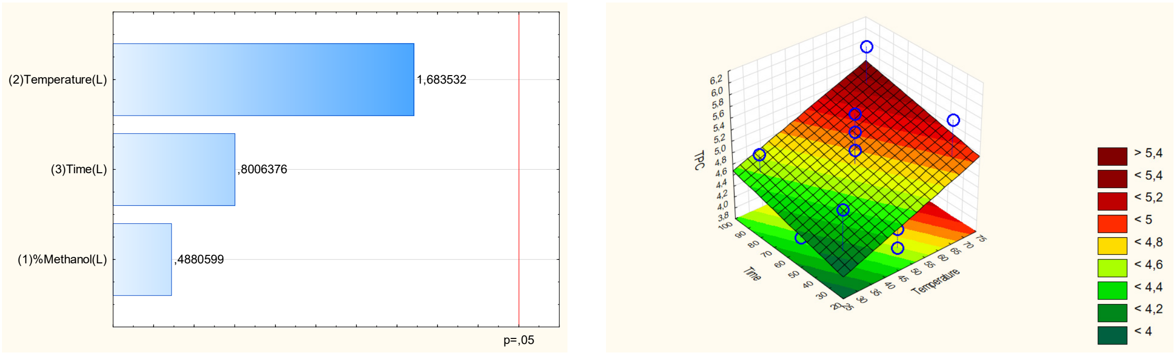

(a) (b)

Figure S1. Statistical analysis for total phenolic content in extracts E1-E9: (a) Pareto plot of standardized effects for total phenolic content in extracts E1-E15; (b) Response surface plots presenting the dependence of extraction temperature and time on the total phenolic content for constant methanol content in the extraction mixture at level 60%.

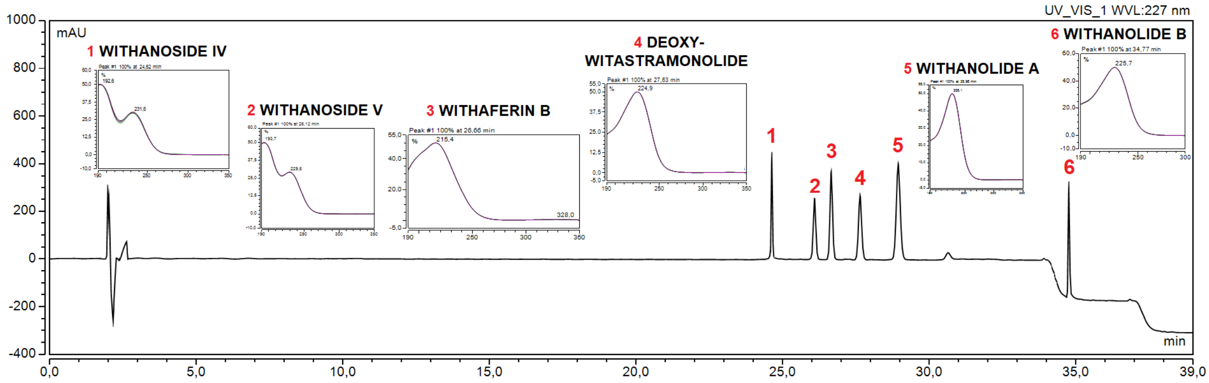

Figure S2. HPLC chromatogram.

Table S1. Validation parameters of HPLC method

Parameter	Withanoside IV	Withanoside V	Withaferin B	Deoxywita-stamonolide	Withanolide A	Withanolide B
Linearity: y = ax + b						
$a \pm S_a$	0.001 ± 0.001	0.015 ± 0.001	0.015 ± 0.001	0.041 ± 0.003	0.039 ± 0.001	0.022 ± 0.001
$b \pm S_b$	insignificant (α=0.05)	insignificant (α=0.05)	insignificant (α=0.05)	insignificant (α=0.05)	insignificant (α=0.05)	insignificant (α=0.05)
Correlation coefficient (r)	0.998	0.999	0.991	0.997	0.953	0.998
Range of linearity [mg/mL]	0.88–8.80	0.80–8.00	0.44–4.40	0.40–4.00	0.56–5.60	2.00–20.00
Intra-day precision, RSD (<5% required) = repeatability						
The lowest concentration	3.67	0.90	1.11	3.07	0.85	0.48
The middle concentration	0.44	0.64	2.46	0.38	1.81	1.31
The highest concentration	3.20	2.23	4.26	0.40	3.83	0.40
Limit of detection (LOD) [μg/mL]	122.72	13.22	63.62	62.18	309.27	268.74
Limit of quantification (LOQ) [μg/mL]	371.88	40.06	192.78	188.42	937.19	814.38

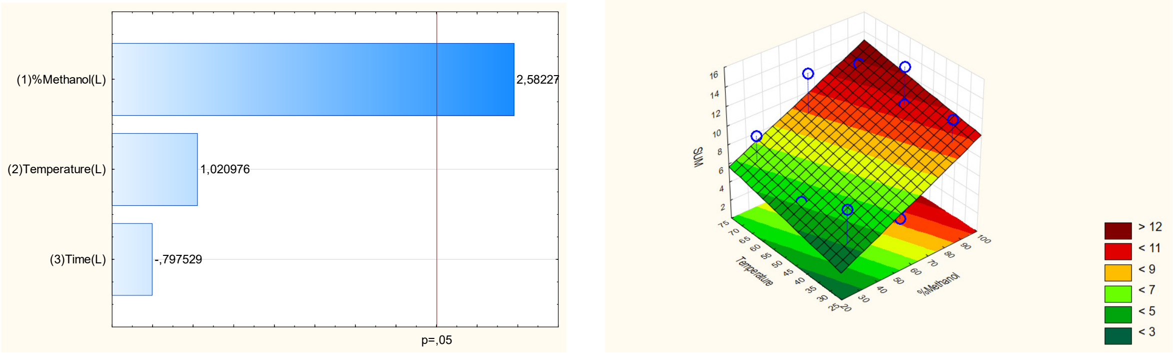

(a) (b)

Figure S3. Statistical analysis for withanolides content in extracts E1-E15: (a) Pareto plot of standardized effects for withanolides content in extracts E1-E15; (b) Response surface plots presenting the dependence of methanol content in the extraction mixture and extraction temperature on withanolides content for constant time at level 60 minutes.

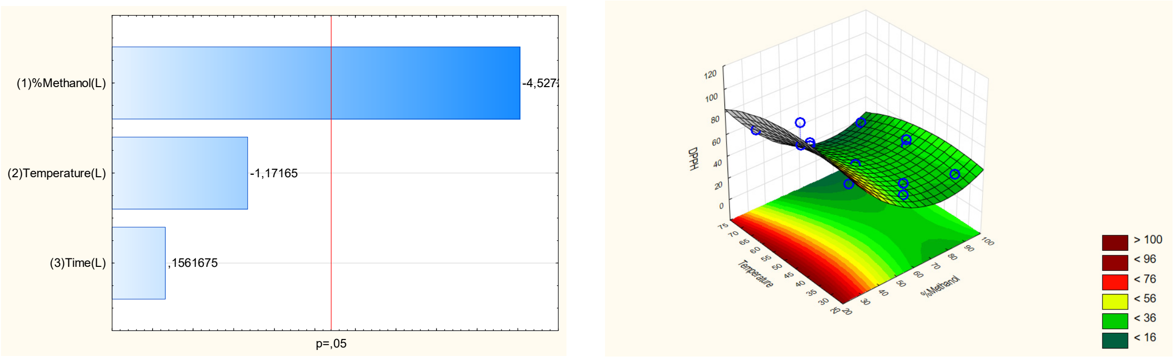

(a) (b)

Figure S4. Statistical analysis for antioxidant activity of extracts E1-E15 measured by DPPH method: (a) Pareto plot of standardized effects for antioxidant activity; (b) Response surface plots presenting the dependence of methanol content in the extraction mixture and extraction temperature on antioxidant activity of extracts for constant time at level 60 minutes.

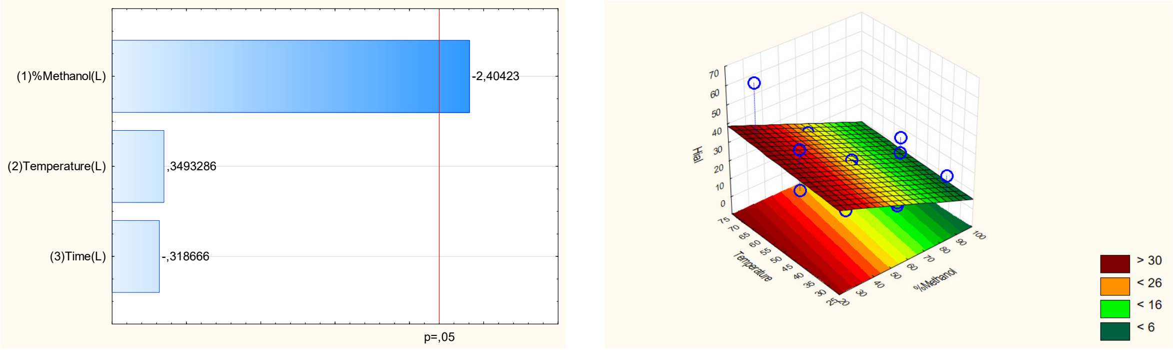

(a) (b)

Figure S5. Statistical analysis for anti-inflammatory activity of extracts E1-E15: (a) Pareto plot of standardized effects for anti-inflammatory activity; (b) Response surface plots presenting the dependence of methanol content in the extraction mixture and extraction temperature on anti-inflammatory activity of extracts for constant time at level 60 minutes.

Table S2. Correlation matrix for phytochemical activities

Variable	TPC	Withanolides	DPPH	Hyal
TPC	1	-0.2212	-0.5613	-0.3819
Withanolides	-0.2212	1	-0.3949	-0.2750
DPPH	-0.5613	-0.3949	1	0.5209
Hyal	-0.3819	-0.2750	0.5209	1

Statistically significant relationships are marked in red

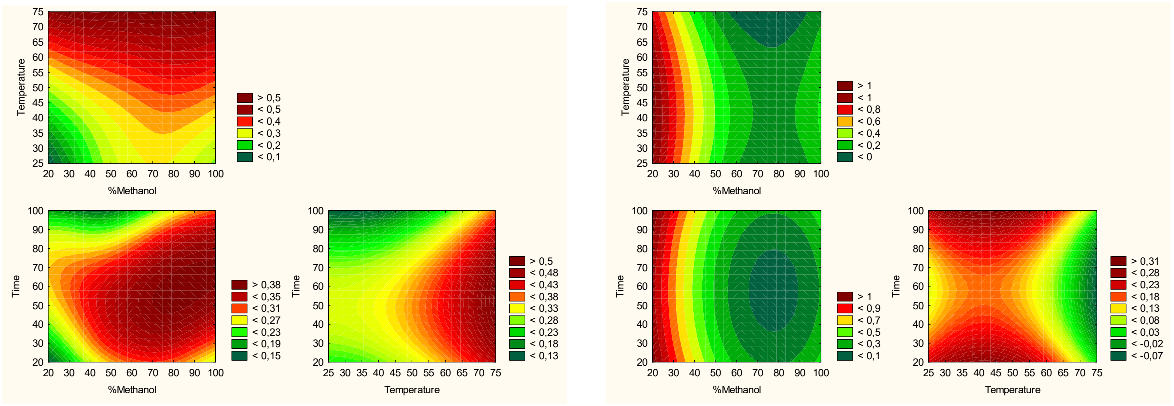

(a) (b)

Figure S6. Prediction of the optimization model for obtaining extracts based on effect with positive sign like TPC, withanolides content and hyaluronidase (a) and those with negative sing like DPPH assay (b).

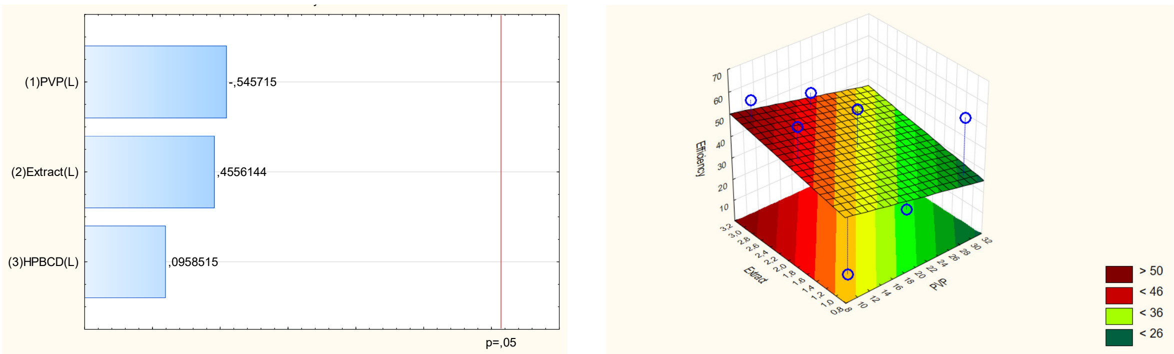

(a) (b)

Figure S7. Statistical analysis for process efficiency: (a) Pareto plot of standardized effects for viscosities of the prepared solutions for electrospinning; (b) Response surface plots presenting the dependence of PVP and extract content on the efficiency of electrospinning process for HPβCD content at level 1.

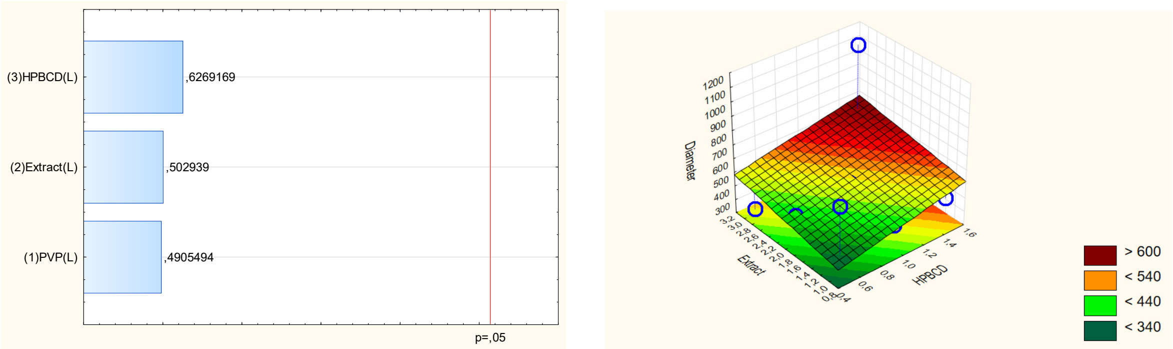

(a) (b)

Figure S8. Statistical analysis for diameter of nanofibers: (a) Pareto plot of standardized effects for diameter of nanofibers F1-F9; (b) Response surface plots presenting the dependence of HPBCD and extract content on the nanofibers' diameter for constant PVP content at level 20%.

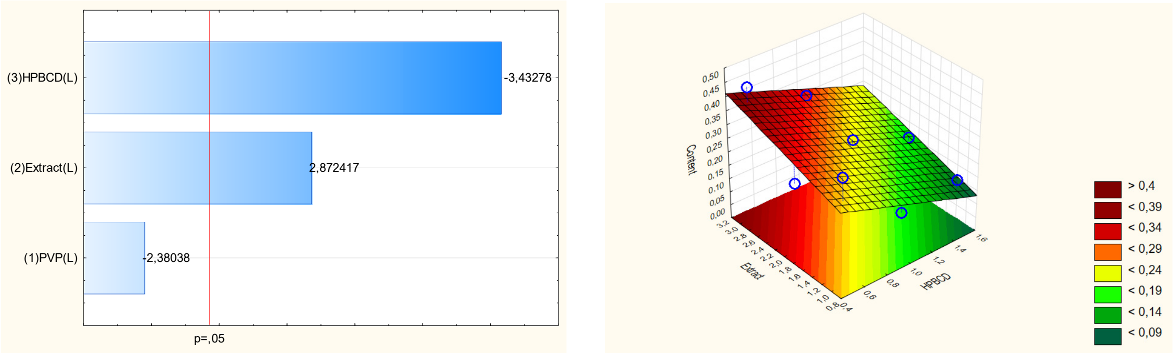

(a) (b)

Figure S9. Statistical analysis for withanoside IV content: (a) Pareto plot of standardized effects for withanoside IV content in nanofibers F1-F9; (b) Response surface plots presenting the dependence of HPβCD and extract content on the withanoside IV content for constant PVP content at level 20%.

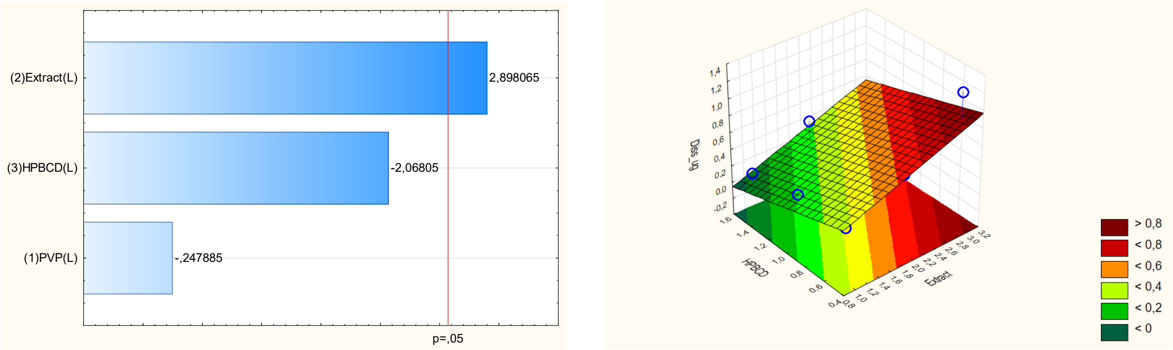

(a) (b)

Figure S10. Statistical analysis for withanoside IV dissolution: (a) Pareto plot of standardized effects for withanoside IV dissolution in 5 minutes from nanofibers F1-F9; (b) Response surface plots presenting the dependence of extract and HPβCD content on the withanoside IV dissolution for constant PVP at level 20%.

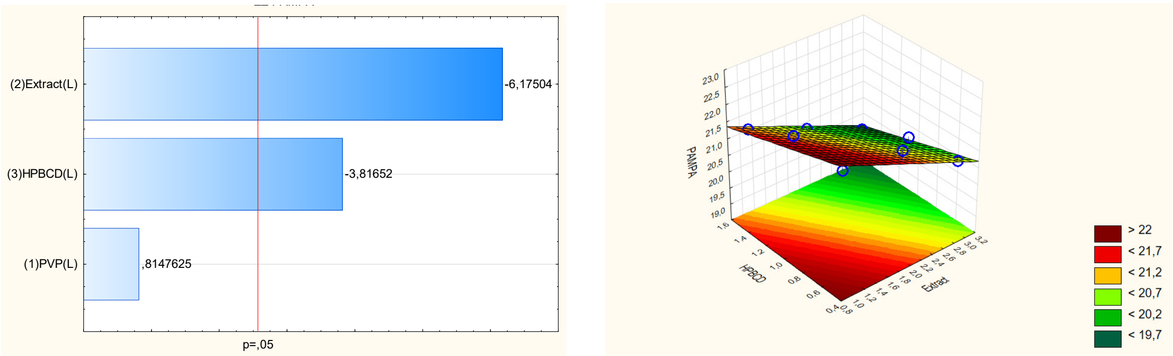

(a) (b)

Figure S11. Statistical analysis for withanoside IV permeability: (a) Pareto plot of standardized effects for withanoside IV permeability from nanofibers F1-F9; (b) Response surface plots presenting the dependence of extract and HPβCD content on the withanoside IV permeability for constant PVP content at level 20%.

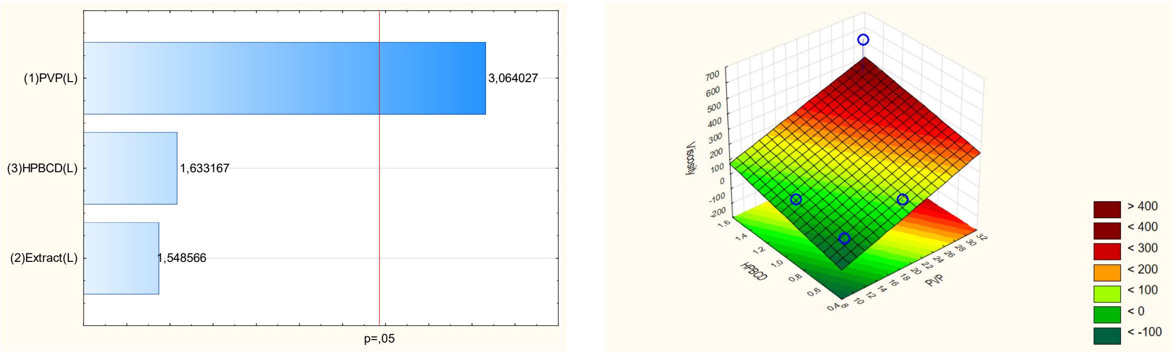

(a) (b)

Figure S12. Statistical analysis for component of mucoadhesion: (a) Pareto plot of standardized effects for mucoadhesive properties of nanofibers F1-F9; (b) Response surface plots presenting the dependence of PVP and HPβCD content on the component of mucoadhesion for constant extract content at level 2 ml.

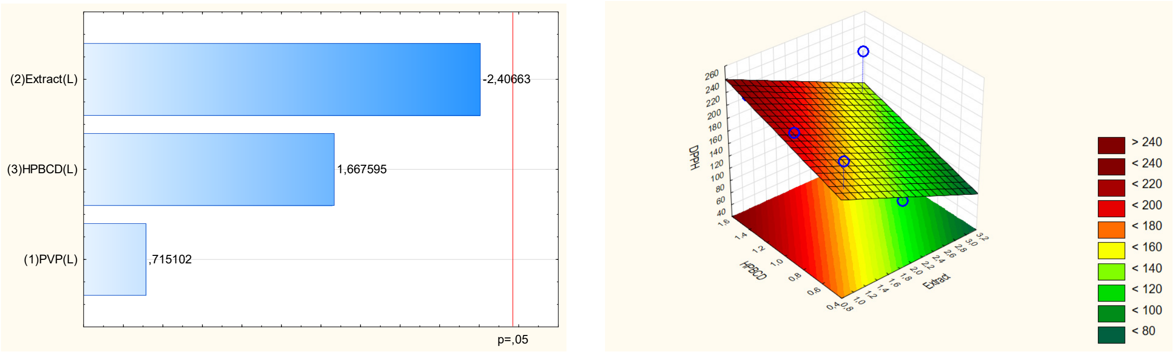

(a) (b)

Figure S13. Statistical analysis for antioxidant activity of nanofibers: (a) Pareto plot of standardized effects for antioxidant activity of nanofibers F1-F9; (b) Response surface plots presenting the dependence of extract and HPβCD content on the antioxidant activity for constant PVP content at level 20%.

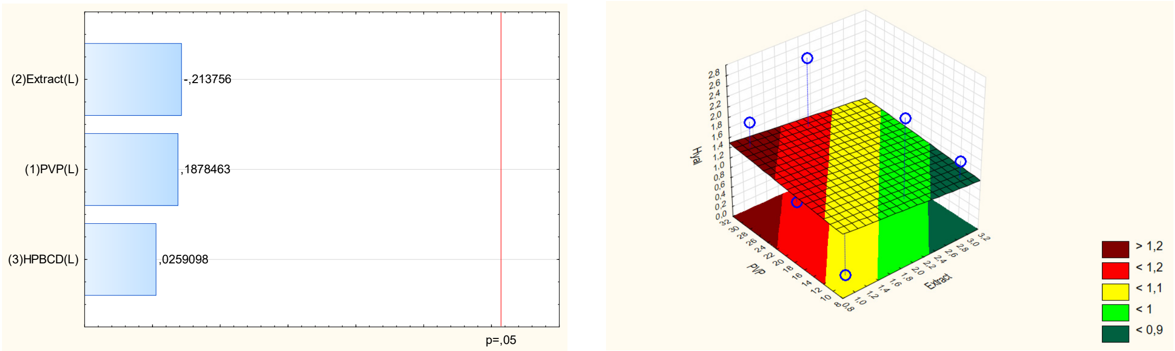

(a) (b)

Figure S14. Statistical analysis for anti-inflammatory activity of nanofibers: (a) Pareto plot of standardized effects for anti-inflammatory activity of nanofibers F1-F9; (b) Response surface plots presenting the dependence extract and PVP content on the anti-inflammatory activity for constant HPβCD content at level 1.

Table S3. Correlation matrix for nanofibers properties

Variable	Efficiency	Diameter	Content of withanoside IV	Dissolution of withanoside IV	PAMPA	Component of mucoadhesion	DPPH	Hyal
Efficiency	1,0000	-0,7063	0,0263	0,3395	-0,1497	-0,4538	-0,5810	0,4331
Diameter	-0,7063	1,0000	-0,0337	-0,3659	-0,3553	0,7514	0,5123	-0,5522
Content of withanoside IV	0,0263	-0,0337	1,0000	0,7270	-0,2368	-0,2769	-0,5507	-0,4326
Dissolution of withanoside IV	0,3395	-0,3659	0,7270	1,0000	-0,2945	-0,1277	-0,8110	0,1021
PAMPA	-0,1497	-0,3553	-0,2368	-0,2945	1,0000	-0,4282	0,2616	0,2954
Component of mucoadhesion	-0,4538	0,7514	-0,2769	-0,1277	-0,4282	1,0000	0,3569	-0,2356
DPPH	-0,5810	0,5123	-0,5507	-0,8110	0,2616	0,3569	1,0000	-0,3476
Hyal	0,4331	-0,5522	-0,4326	0,1021	0,2954	-0,2356	-0,3476	1,0000

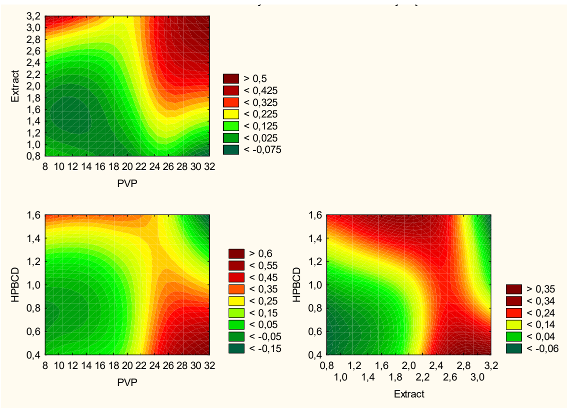

(a)

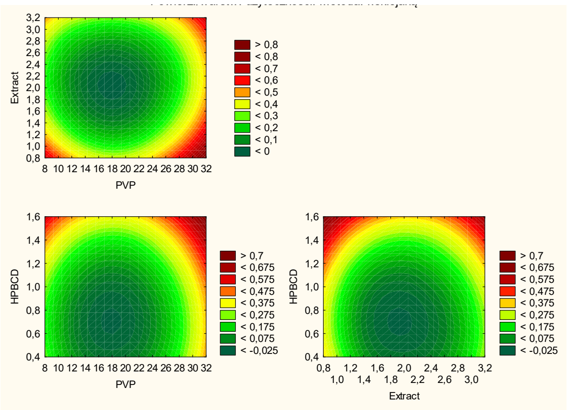

(b)

Figure S15. Prediction of the optimization model for obtaining extracts based on effects with positive sign like process efficiency, withanoside IV content, dissolution and permeability, Hyal as well as component of mucoadhesion (a) and this with negative sing like nanofibers diameters and DPPH (b).
